# Acknowledgment to Reviewers of *Marine Drugs* in 2021

**DOI:** 10.3390/md20020107

**Published:** 2022-01-28

**Authors:** Marine Drugs Editorial Office

**Affiliations:** MDPI AG, St. Alban-Anlage 66, 4052 Basel, Switzerland

Rigorous peer-reviews are the basis of high-quality academic publishing. Thanks to the great efforts of our reviewers, *Marine Drugs* was able to maintain its standards for the high quality of its published papers. Thanks to the contribution of our reviewers, in 2021, the median time to first decision was 12 days and the median time to publication was 29 days. The editors would like to extend their gratitude and recognition to the following reviewers for their precious time and dedication, regardless of whether the papers they reviewed were finally published:
Abdelfattah, MohamedLittlefield, Bruce A.Abdel-Latif, Hany M. R.Liu, BinAbdollahi, MehdiLiu, Shing-HwaAcquaviva, RosariaLlewellyn, CaroleAdachi, KohsukeLoiseau, NicolasAdamczyk-Grochala, JagodaLoll, BernhardAdhikary, TillLongoni, Silvia StefaniaAgòcs, AttilaLópez, Lucía MéndezAhmed, Atallah F.López-López, ManuelAigle, BertrandLorant, AnneAirs, Ruth LouiseLordan, RonanAkiyama, MasahiroLowell, AndrewAlarcón López, Francisco JavierLu, Wen-ChienAlbano, FrancescoLuis, AnaAlbericio, FernandoLuiz, Rodrigo CabralAlbini, AdrianaLundholm, NinaAleu, JosefinaMaceiras, RocíoAllen, ScottMaciej, StrzemskiAlonso, Juan RubioMadhukar, BurraAltemimi, AmmarMaduro, MorrisAlvarez, Ana I.Maeda, HayatoÁlvarez, Enrique DomínguezMagarlamov, Timur Yu.Alvariño, RebecaMaggi, Leonard B.Aminin, DmitryMagriotis, Plato A.Amoutzias, GrigorisMakarska-Bialokoz, MagdalenaAmsler, CharlesMakhutova, OlesiaAna, Borrego-SánchezMalara, AlessandroAnastyuk, Stanislav D.Malfa, Giuseppe AntonioAndó, IstvánMalik, AdeelAndrianasolo, EricMallamaci, RosannaAngelopoulou, AthinaManabe, YukiAnniballi, FabrizioMancini, InesAntunes, JoanaMándi, AttilaAntunes, JorgeMangesh, HarishApicella, AntonioMangoni, AlfonsoAprotosoaie, Ana ClaraManzo, EmilianoArai, MasayoshiMaramai, SamueleArakawa, OsamuMarcial-Quino, JaimeArellano, Juan B.Marcos, RicardoArisaka, YoshinoriMaresca, MarcArita, MasanoriMarin, Ricardo MallaviaArroba, Ana I.Markopoulos, AnastasiosArteaga, Jesús FernándezMarquez-Rocha, Facundo J.Artyukhin, AlexMarra, AlbertoAsamizu, ShumpeiMartin, NeilAshworth, Matt P.Martínez, Antonio RosalesAtobe, MasakazuMartínez-Álvarez, ÓscarAvila-Román, JavierMartínez-Espinosa, Rosa MaríaAvilés, Francesc XavierMartín-Gil, JesusAvni, DoritMartins, AliceAyala, JuanMartins, RosárioAzam, LaylaMaruyama, HirokoBacchiocchi, SimoneMaskrey, BenjaminBae, Ji-YeongMassiot, GeorgesBae, Tae SooMatos, Ana RitaBaek, Kyung InMatsuura, HiroshiBaharum, Syarul NataqainMatte, AlessandroBaindara, PiyushMattei, CésarBaker, Mark D.Maurer, StefanieBaker-Austin, CraigMáximo, PatríciaBalen, BiljanaMccall, Laura-IsobelBaltz, RichardMccarthy, PeterBanskota, Arjun H.Mcfadden, SandraBarbaric, MonikaMcmahon, Hilary E. M.Bardají, EduardMcphail, Kerry L.Barh, DebmalyaMediero, AránzazuBarnathan, GillesMeghea, AureliaBarraja, PaolaMehariya, SanjeetBartosz, GrzegorzMekasha, SophanitBasile, LiviaMekinić, Ivana GeneralićBatista, IrineuMelendez-Ortiz, HectorBeedessee, GirishMenchinskaya, Ekaterina S.Behringer, ErikMeslet-Cladiere, LaurenceBeld, JorisMester, Patrick-JulianBello, ClaudiaMicael, JoanaBelluzzi, ElisaMichaud-Soret, IsabelleBelosludtsev, KonstantinMilanova, AneliyaBen-Gigirey, BegoñaMiller, IngridBeppu, FumiakiMillot, MarionBergandi, LoredanaMiloso, MariarosariaBerger, StefanMilthorpe, BruceBerillis, PanagiotisMinato, Ken-IchiroBerrue, FabriceMinkiewicz, PiotrBertrand, SamuelMinnelli, CristinaBezirtzoglou, EugeniaMinto, Robert E.Bharath, Leena P.Miricescu, DanielaBiagini, GiuseppeMiserocchi, GiacomoBiernasiuk, AnnaMitaine-Offer, Anne-ClaireBilancio, AntonioMitchell, MiguelBinda, ElisaMiyanaga, AkimasaBiré, RonelMiyashita, KazuoBirsa, Mihail LucianMlostoń, GrzegorzBissoyi, AkalabyaMoeini, ArashBlanco, JuanMogielnicki, AndrzejBlank, MiriMohammed, AltafBlasiak, JanuszMoldenhauer, MarcusBlazina, MariaMolgó, JordiBlümel, MartinaMolinaro, AntonioBocian, SzymonMoloney, MarkBoggia, RaffaellaMoncalián, GabrielBohara, RaghvendraMonge, ClaireBöhrnsen, FlorianMonteiro, PedroBolbasov, EvgeniyMontero, OlimpioBölcskei, HedvigMorales, SerafinBonaccorsi Di Patti, Maria CarmelaMoreira, Daniel CarneiroBonaventura, RosaMorel-Rouhier, MélanieBondon, ArnaudMori, HirotadaBonfili, LauraMori, TetsushiBontempo, PaolaMorimura, ShigeruBorges Dos Santos, Rui M.Morita, NaokiBorkowska, MonikaMorzycki, Jacek W.Boros, LaszloMoschovi, MariaBortner, Carl D.Mot, AugustinBossowska, AgnieszkaMouga, TeresaBotana, Ana MaríaMourão, Paulo A. S.Botelho, Maria JoaoMozumder, Md. Salatul IslamBotta, BrunoMozzicafreddo, M.Boudreau, Paul D.Mroczek, AgnieszkaBoukouvalas, JohnMudge, Elizabeth M.Boundy, Michael J.Mukai, HidehitoBourgougnon, NathalieMuller, MarcBourguet-Kondracki, Marie LiseMulloy, BarbaraBowden, BruceMunekata, PauloBracco, EnricoMuramoto, KojiBraga, Susana SantosMurata, MichioBravo, Susana B.Murias, MarekBrecker, LotharMurkovic, MichaelBrett, ElizabethMurphy, Patrick J.Brown, Alan B.Muszyński, SiemowitBrown, LindsayMyllykallio, HannuBrownlee, Iain ANagai, HiroshiBruno, AntoninoNagao, KojiBrycki, BogumilNagaoka, IsaoBulet, PhilippeNaghdi, SamiraButu, AlinaNakata, NorioCacciola, FrancescoNam, Sang-JipCalado, Cecília R. C.Namgaladze, DmitryCalado, RicardoNandin-Erdene, MandakhbayarCalcinai, BarbaraNanno, MasanobuCaldara, MarinaNapolitano, AlessandraCalina, DanielaNashmi, RaadCametti, CesareNathanailides, KosmasCampbell, ChristopherNavarro, Juan C.Campera, MarcoNavarro, Macarena SánchezCañada, FranciscoNaviglio, DanieleCapillo, GioeleNedialkov, Paraskev T.Capon, RobNeilson, AndrewCarballeira, Nèstor M.Neumann, JakeCarbonell-Barachina, AngelNeureiter, DanielCarda, MiguelNeut, ChristelCardoza, Rosa ElenaNg, Tzi BunCarmeli, ShmuelNicolau, ElodieCaruso, EnricoNishimura, ShinichiCaruso, GabriellaNitulescu, MihaiCarvalho, FatimaNoble, Peter A.Carvalho, PauloNociari, MarceloCasalino, ElisabettaNoda, MasafumiCasaril, AngelaNorte, ManuelCasas, FrançoisNorton, RaymondCasertano, MarcelloNovelli, AntonelloCastro, José Javier FernándezNtaikou, IoannaCastro-Muñoz, RobertoNugraha, BramastaCatani, MartinaOchiuz, LacramioaraCatanzaro, ElenaOczkowski, MichałCavallaro, GiuseppeOdintsova, N. A.Caviglia, Gian PaoloOh, Chang-MyungCerra, BrunoOh, Dong-ChanĆetković, HelenaOh, HyuncheolChaikuad, ApiratO'Harte, FinbarrChalamaiah, MeramOhshiro, TakashiChandra-Hioe, Maria VeronicaOhta, ShinjiChang, Long-SenOkamura, YoshikoChao, Louis KuopingOkino, TatsufumiCharoensutthivarakul, SitthivutOkumura, KoCharusanti, PepOkunade, Adewole L.Chatzidoukas, ChristosOlejnik, AnnaChen, Hsin-YuanOlichon, AurélienChen, JiezhongOliveira, AnaChen, Shuen-EiOng, Eng ShiChenais, BenoitOniszczuk, AnnaChénais, BenoitOoi, Chien WeiCheng, Chih-YuOpatz, TillCheong, Kit LeongOprea, OvidiuChernikov, OlegOpydo-Chanek, MałgorzataChevrot, RomainOrefice, IdaChianese, GiuseppinaOrfali, RahaChiavaroli, AnnalisaOrlandini, ElisabettaChimienti, GiovanniOrtiz-Sánchez, ElizabethChizhov, Alexander O.Orvalho, SandraChlichlia, KaterinaOshiro, NaomasaChoi, ChanghoonOtsuki, TakemiChoi, Jong-IlOvchinnikova, Tatiana V.Chyau, Charng-CherngOzaltin, KadirCiccarelli, RenataOzeki, TetsuyaCicero, ArrigoOzeki, YasuhiroCichero, ElenaPadrão, JorgeCiesielska, AnnaPalenzuela, José AntonioCieślińska, AnnaPalijan, GoranCirrincione, GirolamoPallio, GiovanniClair, James LaPalumbo, AnnaClericuzio, MarcoPalumbo, CarlaColavito, Sierra A.Pampalakis, GeorgiosCollier, JackiePanaro, Maria AntoniettaConlon, MichaelPapadimitriou, TheodotiContaldo, MariaPapini, EmanueleCoombs, MelaniePark, Ah-MeeCord-Landwehr, StefanPark, ChulhwanCorral, PaulinaPark, Jin-SooCorreia-Da-Silva, MartaParsons, PeterCorsaro, Maria MichelaPasqualetti, MarcellaCosta, Pedro M.Passalacqua, KarlaCostantino, ValeriaPateiro, MirianCosta-Pinto, Ana RitaPatruno, AntoniaCotas, JoãoPavao, Mauro SergioCoustau, ChristinePavic, AleksandarCrossman, LisaPavlov, AtanasCrüsemann, MaxPawlak, AleksandraCueto, MercedesPearce, CedricCui, QiuPearce, NorrieCutignano, AdelePecorelli, AlessandraCuypers, BartPedersen, Kim B.Czernel, GrzegorzPelin, MarcoD'Agostino, Paul M.Pentak, DanutaDahiya, RajivPepi, MilvaDahong, LiPerdih, AndrejD'Ambra, IsabellaPereira, DavidDamiano, FabrizioPereira, FlorbelaDamkaci, FehmiPereira, José AugustoDanac, RamonaPereira, LeonelDaniela, AilincaiPereira, Patrícia AlexandraDansette, PatrickPescitelli, GennaroDarie-Nita, Raluca NicoletaPeter, MartinD'Auria, Maria ValeriaPfeiffer, IlonaDavis, David A.Phan, Chin-SoonDe Filippis, BarbaraPhelix, Clyde F.De Francesco, FrancescoPhilippopoulos, Athanassios I.De Los Reyes, CarolinaPhilips, NeenaDe Lourdes Pereira, MariaPi, JiangDe Mieri, MariaPiazza, StefanoDe Vendittis, EmmanuelePickett, AndyDegl'innocenti, DonatellaPickova, JanaDel Pulgar, Sofia PerezPiecuch, AgataDella Sala, GerardoPiestansky, JurajDemchuk, OlehPiggott, AndrewDemori, IlariaPilkington, LisaDeslandes, EricPina-Perez, Maria ConsueloDesmaële, DidierPinheiro, JoaquinaDevkota, Hari PrasadPinto, Diana CláudiaDewapriya, PradeepPires, CarlaDi Bari, LorenzoPisklak, Dariusz MaciejDi Cesare Mannelli, LorenzoPitucha, MonikaDi Gaetano, SoniaPlastina, PierluigiDi Lorenzo, FrancescoPlatta, HaraldDiacon, AurelPletzer, DanielDias, Fernanda F. G.Pohnert, GeorgDingley, Andrew J.Poku, Rosemary A.Dinica, Rodica-MihaelaPolčic, PeterDinos, GeorgiosPolticelli, FabioDiţu, Lia MaraPons, Daniel GabrielDiverdi, JosephPoquet, LaureDobrzynski, PiotrPotaczek, Daniel P.Doi, TakayukiPoulin, RemingtonDolashka, PavlinaPozharitskaya, OlgaDomalik-Pyzik, PatrycjaPrada, JustinaDomingues, RosarioPradhan, Bhola ShankarDomínguez, HerminiaPriel, AviDomínguez-Álvarez, EnriquePrieto, MiguelDomonkos, IldikóPrieto, Miguel A.Dorantes-Aranda, Juan JoséPrieto, Miguel ÁngelDoroftei, BogdanPrim, DamienDoupnik, Craig A.Prisic, SladjanaDrabowicz, JózefProcopio, Francesco AndreaDu, DeguoPucciarelli, SandraDuan, DelinPuillandre, NicolasDufossé, LaurentPuizina, JasnaDumic, JerkaQuaglino, DanielaDurmus, NedimQuaglio, DeborahDuszka, KalinaQuintas, AlexandreDutartre, PatrickRabbani, GulamDutertre, SebastienRacovita, StefaniaDutertre, SébastienRadulescu, CristianaDutot, MélodyRadwan, MohamedDvoretsky, Alexander G.Rafael, RobainaDyshlovoy, Sergey A.Ragnarsson, LottenDzidic, AlenRahman, M. AzizurDzunkova, MariaRai, AkhileshEfremenko, ElenaRaja, RathinamEinarsson, HjorleifurRamazzotti, MatteoEl Mourabit, HaquimaRapeanu, GabrielaElinder, FredrikRateb, MostafaEllinger, BernhardRaudonė, LinaEl-Sheekh, MostafaRaugei, GiovanniEngelmann, PeterRavi, Rama S.Enguita, Francisco J.Ravindran, RajeevEscrig, Nuria CabedoRawski, MateuszEsposito, RobertaRebollo-Hernanz, MiguelEsteves, Ana CristinaRebuffat, SylvieEttayapuram-Ramaprasad, Azhagiya SingamRegina, Giuseppe LaFabiszewska, AgataReis, CristianoFakira, Amanda K.Reis-Costa, PedroFalcó, AlbertoRemias, DanielFarjon, JonathanRevuelta, JuliaFedorov, Alexey N.Reyes, Carolina PérezFelgueiras, HelenaReyes, FernandoFeng, Sheng-WeiReynisson, JóhannesFernández-Acero, Francisco JavierRhee, Jae-SungFernandez-Lafuente, RobertoRho, Jung-RaeFernando, I. P. ShanuraRicciardi, Maria RosariaFernando, Ilekuttige Priyan ShanuraRiccio, GennaroFerreira, AntonioRimondini, LiaFigadere, BrunoRoberti, RobertaFigueroa, Félix L.Rochefort, Gael Y.Figuerola, BlancaRodrigues, Márcio JoséFilip-Psurska, BeataRodrigues, Maria JoãoFinetti, FedericaRodriguez, IsabelFitton, Janet HelenRodríguez, JaimeFlorean, CristinaRodríguez-Yoldi, María JesúsFlores, CintiaRoje, MarinFlórez-Fernández, NoeliaRok, JakubFogacci, FedericaRomagnoli, RomeoFontana, AngeloRomano, GiovannaFontana, FabrizioRosengren, JohanFoubelo, FranciscoRoss, Ian L.Fouillen, LaetitiaRossi, ClaudioFraga-Corral, MariaRoth, ChristianFruit, CorinneRóżalska, BarbaraFujita, MasakiRozza, ArianeFuruita, HirofumiRudolf, EmilFuwa, HaruhikoRuml, TomasGabrielli, Maria GabriellaRuocco, NadiaGăină, Luiza IoanaRussell, FraserGaleazzi, RobertaRyabchikova, Elena I.Galicka, AnnaRye, Philip D.Gallo, MonicaRyffel, BernhardGalvagni, FedericoRyu, BomiGama, SofiaSabot, CyrilleGanesan, A.Sadamoto, HisayoGarbayo, InésSadovskaya, IrinaGarcia, Jose PrietoSaez, CarmenGarcia, RonaldSaez, Jose ColonGarcia-Jimenez, PilarSáez-Casado, María IsabelGardikis, KonstantinosSafavi, MalihehGarzoli, StefaniaSaha, SubhasishGavenonis, JasonSaini, Ramesh KumarGebauer, JanSakai, RyuichiGęgotek, AgnieszkaSakayori, NobuyukiGenovese, GiuseppaŠalić, AnitaGentile, Maria TeresaSalim, AngelaGerdol, MarcoSalinas, EvaGiampietro, LetiziaSalomon, ChristineGiastas, PetrosSalvador-Reyes, LilibethGiordano, DanielaSalzano, Anna MariaGiordano, GuidoSamaja, MicheleGiordano, PaolaSampedro, DiegoGiráldez, InmaculadaSanchez-Amat, AntonioGisch, NicolasSanchez-Ruiz, AntonioGiudice, Angelina LoSancho-Vaello, EneaGiuliani, MariaSangadala, SreedharaGiuliano, MichelaSanjeewa, Kalu Kapuge AsankaGlukhov, EvgeniaSansoe, GiovanniGolomazou, EleniSansone, AnnaGomes, Carlos MartinsSantabarbara, StefanoGomes, Joao R.Santamaria, RamonGomes, NelsonSarabia, FranciscoGómez-Pinchetti, Juan LuisSaratale, Rijuta GaneshGomez-Pinilla Cerezo, Maria NatividadSaravanakumar, KandasamyGomez-Pinilla, Pedro J.Šarkanj, BojanGonçalves, Ana M. M.Sastre, LeandroGonçalves, MicaelSatake, MasayukiGonkowski, SlawomirSatish, LakkakulaGonzález-Minero, Francisco JoséSato, ShigeruGonzalez-Vicente, AgustinSaul, NadineGorbushin, AlexanderSaurav, KumarGórska, AgataSayed, AhmedGórska, SabinaScarfì, SoniaGorzelanny, ChristianSchikov, AlexanderGoti, AndreaSchlein, ChristianGrasselli, ElenaSchmid, JochenGrimaldi, AnnalisaSchrama, DeniseGrimm, MarcusSchroeder, ChristinaGroff, JosephScocchi, MarcoGrovel, OlivierScott, Nicola Jean AgnesGrzesiak, JakubSeale, Lucia A.Gubitosa, JenniferSearcey, MarkGuella, GrazianoSeca, Ana M. L.Guo, ChangjunSeca, Ana Maria LoureiroGuriec, NathalieSedlic, FilipGustafson, KirkSeguí Gil, LucíaGutierrez, Mar FernandezSeijas Vázquez, Julio A.Guzmán, EduardoSela, ShlomoGuzmán-Ruiz, RocíoSelwood, AndyGuzow-Krzemińska, BeataSemenycheva, Ludmila L.Hachero, IsmaelSeo, Ji-HunHallegraeff, Gustaaf M.Serralheiro, Maria LuísaHamashima, YoshitakaSeto, ShigekiHanas, JayShaala, Lamiaa A.Hanif, NovriyandiSham, Yuk Y.Hannan, Md. AbdulSharma, KavitaHansen, EspenSharma, NikitaHantke, KlausShavandi, AminHaq, MonjurulShave, StevenHardy, Rowan S.She, Zhi-GangHarrison, Paul H. M.Shene, CarolinaHarvey, JoanneSheu, Jyh-HorngHarvey, Patricia J.Shidoji, YoshihiroHarvey, PetaShigemura, YasutakaHasan, ImtiajShikov, AlexanderHassouna, Amira AhmedShikov, Alexander N.Haverkamp, Richard G.Shin, Hee JaeHayashi, KyokoShin, JongheonHayashida, KenjiShin-Ya, KazuoHegazy, Mohamed-Elamir F.Shnyder, SteveHernández-Daranas, AntonioSiebert, Hans-ChristianHernandez-Prieto, Miguel A.Sil, SusmitaHeymann, DominiqueSilachev, DenisHicks, LeslieSilchenko, Alexandra S.Higashi, NobuakiSilipo, AlbaHirabayashi, JunSilva, AuroraHoffman, AngelaSilva, JoanaHojan, KatarzynaSilva-Abreu, MarcelleHomaeigohar, ShahinSilvestre, SamuelHorakova, OlgaŠindlerová, LenkaHotos, George N.Sing, Swee LeongHou, Chih-YaoSingh, JonathanHrabal, RichardSingla, BhupeshHsu, Fong-FuSionov, EdwardHuang, Chun-YungSiopa, FilipaHuang, JinlingSipkema, DetmerHuerta-Yépez, SaraSivanesan, IyyakkannuHughes, Christopher V.Skorko-Glonek, JoannaHung, AndrewSkouta, RachidHwang, Bang YeonSkropeta, DanielleIatridi, ZacharoulaSlamova, KristynaIbrahim, Mohamed AliSmerilli, AriannaIbrahimi, ManarSmith, G. JasonImperatore, ConcettaSmith, KerryImperatore, RobertaSöderhäll, KennethInagaki, HidetoshiSogawa, NorioInestrosa, Nibaldo C.Solano, FranciscoInuzuka, ToshiyasuSolovchenko, AlexeiIqbal, Hafiz M. N.Song, FuhangIsaeva, Marina P.Song, Myoung ChongIshii, TakahiroSong, Myoung-ChongIslam, SalmanSorensen, JohnItoh, TakafumiSoreq, HermonaItoi, ShiroSoriano-Romaní, LauraIwasaki, ArihiroSoriano-Ursúa, Marvin A.Izzo, IreneSosa, SilvioJakas, AndrejaSosio, MargheritaJang, KyuyunSosnowska, KatarzynaJanowski, MiroslawSousa, Maria JoãoJarończyk, MałgorzataSousa, SérgioJassbi, AmirrezaSovadinova, IvaJembrek, Maja JazvinšćakSowa, IreneuszJenner, RonaldSpanò, NancyJenssen, HåvardSperandeo, PaolaJeong, Se KyooSpurio, RobertoJerković, IgorStanciuc, Ana MariaJiang, GuangdeStändker, LudgerJimbo, MitsuruStefan, SimmJimenez, CarlosStefaniuk, DawidJiménez, CarlosStejskal, VlastimilJiménez-Tenorio, ManuelStoskopf, MichaelJin, JunyanStoyneva-Gärtner, MayaJoão, CotasStringer, Damien N.João, SilvaStringham, James M.Jones, ChrisStrøm, Morten B.Jones, Meriel G.Strzelecka-Kiliszek, AgnieszkaJoo, Hong-GuStuart, David T.José-Manuel, Leao MartinsStudzinska-Sroka, ElzbietaJoyce, Susan A.Sugahara, TakuyaJu, JianhuaSugimoto, SachikoJubeau, SébastienSulej, JustynaJung, Jee-HyungSullivan, RyanJurj, Ancuţa MariaSun, RamonKadokawa, Jun-IchiSurup, FrankKafarski, PawelSuzuki, IwaneKafarski, PawełSzaleniec, MaciejKajihara, HiroshiSzekalska, MartaKalinin, VladimirSzkaradkiewicz, AndrzejKalinin, Vladimir I.Tabudravu, Jioji N.Kaminski, KamilTagliazucchi, DavideKaminski, Tomasz W.Taguchi, KenseiKamio, MichiyaTailhades, JulienKanda, TatsuoTakaichi, ShinichiKang, Jeong-HunTakashige, KashimotoKarayannakidis, PanagiotisTakashima, ShigeoKaruppan, Mohan Kumar MuthuTakeshita, SatoshiKasheverov, IgorTalens-Perales, DavidKashman, YoelTam, Roger Y.Kasten-Jolly, JaneTamarit, DanielKaszowska, MartaTamkovich, SvetlanaKatikou, PanagiotaTampucci, SilviaKato, NobutakaTanaka, JunichiKatsuta, ErikoTanaka, NaonobuKatsuyama, YoheiTang, HaoyuKawakami, YukiTareq, Fakir ShahidullahKawamura, AkiraTava, AldoKazakova, Oxana B.Téletchéa, StéphaneKelly, Claire M.Teodoro, JoãoKelly, MichelleTeotia, Arun KumarKem, WilliamTeragawa, HirokiKempken, FrankTersariol, Ivarne L. S.Kerch, GarryTeusch, NicoleKeyzers, RobThomas, Eric JimKeyzers, RobertThomas, OlivierKhan, FazlurrahmanTilocca, BrunoKhan, Mohd SajidTimoshenko, Alexander V.Khotimchenko, Maxim Y.Tiscar, Pietro G.Ki, Jang-SeuTomás, Juan M.Kikukawa, HiroshiTomaszewska, EwaKim, Chu-YoungTommonaro, GiuseppinaKim, EuikyungTonello, FiorellaKim, Hak JunTrček, JanjaKim, HyesunTrincone, AntonioKim, JinsikTrofimov, Aleksei V.Kim, Se-KwonTrojanowicz, BoguszKim, Young-SangTrombetta, Maria FedericaKimura, Ken-IchiTroselj, Koraljka GallKinnel, RobinTsakovska, IvankaKishimura, HidekiTseng, Chih-HuaKiuru, PaulaTsironi, FannyKlar, AgnesTuvikene, RandoKleier, Daniel A.Tziveleka, Leto-AikateriniKlettner, AlexaUcciferri, ClaudioKlewicka, ElzbietaUeno, AkioKO, Kam MingUeno, MikinoriKobayashi, DaisukeUrban, PhilippeKochneva, GalinaVafidis, DimitrisKojima-Yuasa, AkikoVaiano, FabioKokoulin, Maxim S.Vale, PauloKoksharova, Olga A.Van Wijk, Rob ChristiaanKolesnikova, Sophia A.Varna, MarianaKolobov, Alexandr AlexandrovichVarvaresou, AthanasiaKomaki, HisayukiVaughan, MelKomba, ShiroVavitsas, KonstantinosKorolkova, YuliyaVázquez-Tato, M. PilarKorzhikova-Vlakh, EvgeniaVázquez-Ucha, Juan C.Kos, BlaženkaVega-Holm, MargaritaKose, AyseVelázquez-Campoy, AdrianKoshino, HiroyukiVeranic, PeterKostakis, IoannisVezzi, AlessandroKostyuk, VladimirVicente, Filipa A.Kot, Anna MariaVicente, FranciscaKotake-Nara, EiichiVickery, KarenKotańska, MagdalenaViguera, Ana RosaKotoku, NaoyukiVijayaanand, Arokia MariyadossKouokam, Joseph CalvinVijayanand, Arokia MariyadossKoutelidakis, Antonios E.Vilariño, NataliaKoval, OlgaVilchez, CarlosKozakiewicz, AnnaVilcinskas, AndreasKozanecki, MarcinVilla, CarlaKozlov, SergeyVinayak, VandanaKozłowska, JoannaVinogradov, EvgueniiKozłowska, JustynaVistoli, GiulioKrokidis, MariosVitale, Rosa MariaKról, EwelinaVochozka, MarekKrólicka, AleksandraVorob'ev, Mikhail M.Krüger, MarcusVostinaru, OliviuKuenze, GeorgWagner, LianeKulminskaya, Anna A.Wagner, LudwigKumar, AnujWang, BoKuryk, LukaszWang, Hui-MinKuryłowicz, AlinaWang, San-LangKushkevych, IvanWang, ShuminKyzas, GeorgeWatanabe, RyuichiKyzas, George Z.Waugh, Mark G.Laatsch, HartmutWeiss, ÉtienneLabes, AntjeWempe, MichaelLadrat, Christine DelbarreWhite, LindseyLaity, Peter R.Wielgosz-Collin, GaetaneLamas, AlexandreWillett, JuliaLan, WenjianWindshügel, BjörnLapaquette, PierreWińska, KatarzynaLaronze-Cochard, MarieWójciak-Kosior, MagdalenaLauder, BobWojtunik-Kulesza, Karolina A.Laurienzo, PaolaWong, Fung-FuhLauro, GianluigiWöstemeyer, JohannesLauzon, Marc-AntoineWu, Chang-JerLavoie, SergeWu, Cheng-TienLeber, ReginaYamasaki, MasaoLebreton, JacquesYamashita, AtsuyaLeclère, LucasYan, NingLee Chang, Kim JyeYanagihara, AngelLee, Chia-HungYang, HeejungLee, Dae-SungYasuyuki, NogataLee, HyeyoungYen, Chia-HungLee, Hyi-SeungYongsawatdigul, JirawatLee, I-TaYoo, KeunjeLee, Jun SikYoon, In-SooLee, Pyeong ChunYoon, SikLee, Sang KookYotsu-Yamashita, MariLee, Seung-HongYousefi, MortezaLee, YunkyoungYoussef, DiaaLengyel, ImreYurchenko, EkaterinaLephart, Edwin D.Zabetakis, IoannisLeshchenko, Elena V.Zaitsev, AlekseyŁęska, BogusławaZamyatina, AllaLetsiou, SophiaZárate, RafaelLewicki, SławomirZaravinos, ApostolosLewinska, AnnaZastrow, LeonhardLewis, RichardZeuner, BirgitteLeychenko, ElenaZhang, ChangshengLeyton, AllisonZhang, FanLi, FuliZhang, JunzengLi, Min-HuiZhong, FangruiLi, RongfengZhou, ShaoboLi, ZhunZhu, YuyangLiang, Po-HuangZhuravleva, Olesya I.Liaw, Chih-ChuangZouridakis, MariosLienqueo, María ElenaZubía, EvaLin, Chih-ChienZugaza, José LuisLin, Jer-AnZuk, MagdalenaLiou, Je-WenZych, Maria


